# Protective effects of hesperetin on the quality of sperm, apoptosis, lipid peroxidation, and oxidative stress during the process of cryopreservation: An experimental study

**DOI:** 10.18502/ijrm.v19i1.8178

**Published:** 2021-01-25

**Authors:** Jamal Valipour, Sina Mojaverrostami, Beheshteh Abouhamzeh, Masoumeh Abdollahi

**Affiliations:** ^1^Department of Anatomical Sciences, Faculty of Medicine, AJA University of Medical Sciences, Tehran, Iran.; ^2^Department of Anatomy, School of Medicine, Tehran University of Medical Sciences, Tehran, Iran.

**Keywords:** Cryopreservation, Hesperetin, Spermatozoa, Reactive oxygen species.

## Abstract

**Background:**

Hesperetin is a bioflavonoid compound, largely used in Chinese traditional medicine and found plenty in citrus fruits. Hesperetin has beneficial effects against different diseases. The sperm cryopreservation process is a common method that is used in infertility laboratories. It has been reported that during the cryopreservation process, the quality of sperm is significantly reduced.

**Objective:**

To investigate the effect of hesperetin on the quality of human spermatozoa during the cryopreservation process.

**Materials and Methods:**

In this experimental study, 22 sperm sample of normozoospermia men who reffered to the infertility department of the Shariati Hospital (Tehran, Iran) Between October and November 2019 were collect and divided in to three groups as: 1) fresh, 2) control (frozen-thawed group without treatment), and 3) treatment group as frozen-thawed samples supplemented with 20 µM hesperetin. Motility, Viability, morphology, Apoptotic-like changes, intracellular H${}_{2}$O${}_{2}$, intracellular O2${}^{-}$, and lipid peroxidation (LPO) was measured.

**Results:**

Hesperetin treatment during the cryopreservation process of human sperm significantly improved the viability, motility, and morphology rates of the spermatozoa after frozen-thawed process in control group (p < 0.01). In addition, it significantly reduced the reactive oxygen species (ROS) level, LPO level and increased the percentage of viable sperm cells with intact plasma membrane (p < 0.01) after frozen-thawed process.

**Conclusion:**

Hesperetin can improve the quality of human sperm and also protect human sperm against reactive oxygen species, LPO, and apoptosis during the cryopreservation-thawing process.

## 1. Introduction

The cryopreservation process of human sperm was first introduced in the 1960s (1). This process is commonly used in infertility laboratories. This method provides a possibility for fertility maintenance following chemotherapy, radiotherapy, or various surgical procedures. Besides, in cryopreservation process, screening of donated semen that is infected by human immunodeficiency virus (HIV) or hepatitis B virus can be easily performed (2, 3). Formation of intracellular ice crystals, osmotic and thermal stresses during cryopreservation have some adverse effects on sperm quality, particularly sperm motility, after the thawing process, which can damage the cell membrane and leads to cell death (4, 5). The other important adverse effects of freezing-thawing process on sperm are included plasma membrane and mitochondrial membrane damages (6), reduction of mitochondrial function (7), DNA fragmentation (8), and decline in the sperm motility and survival rates (9). It has been indicated that during the freezing-thawing process, the levels of reactive oxygen species (ROS) increases dramatically (10, 11). Although, the small concentration of ROS plays an important role in physiological processes such as sperm capacitation, acrosome reaction, maintaining the fertility ability (12, 13) and fluidity of the membrane (14). However, higher concentrations of ROS causes major sperm functional defects, such as decreasing sperm motility, increasing intracellular enzyme leakage, and damage to sperm DNA (15). On the other hand, overproduction of ROS leads to lipid peroxidation (LPO) which results in reduction of membrane integrity and induces apoptosis (14).

Hesperetin (5,7,30-trihydroxy-40-methoxy flavanone) is a bioflavonoid which is largely used in Chinese traditional medicine (16). It is easily found in citrus fruits as hesperidin (its glycoside form) which is used as a prodrug (17). Dietary hesperidins are deglycosylated to hesperetin by intestinal bacteria prior to absorption (18). Hesperetin has shown to have several positive effects including antioxidant, anti-inflammatory, antimicrobial, anti-carcinogenic and anti-allergic properties (19). Moreover, it is known to acts as a potent antioxidant agent against cadmium-induced testicular damages (20). Moreover, hesperetin has a protective effect against cardiotoxicity induced by doxorubicin (21). Hesperetin also can protect the testis tissue from doxorubicin-induced testicular toxicity (22) and reduced the risk of testicular damages by inhibiting oxidative stress, apoptosis, and inflammation in diabetic rats (23). Hesperetin can be used as a chemopreventive agent against DMH-induced colon cancer (24).

To the best of our knowledge, no study has ever evaluated the effect of hesperetin on mammalian sperm, especially human, therefore the current study aimed at evaluating the protective effects of hesperetin on the quality of human sperm, apoptosis rate, LPO, and oxidative stress levels during human semen cryopreservation.

## 2. Materials and Methods

### Sample collection

Sperm samples from 22 male donors from the infertility department of the Shariati Hospital (Tehran, Iran) Between October and November 2019, whose fertility evaluated using the standard parameters of the semen (concentration, viability, motility, and morphology) according to the World Health Organization (WHO, 2010 guidelines) had been proven, were collected via masturbation inside the sterile containers. Donors were asked to have at least 72 hr of sexual rest. Following the 30-min liquefaction at 37°C and 5% CO${}_{2}$, the semen was evaluated according to the WHO guidelines. To prevent false results, men with the history of chronic illnesses, endocrine disorders, varicocele disease, cigarettes smoking, as well as history of drug use such as vitamins and antioxidants (selenium and zinc) were excluded from the study. Table I presents the characteristics of semen samples from the participant. In the current work, three experimental groups were considered (each semen sample was divided into three groups): First group (fresh group); Second group, frozen-thawed group without treatment with hesperetin (control group); and Third group, frozen-thawed samples were supplemented with 20 µM hesperetin (treatment group), in this group 20 µM hesperetin was added to the semen samples befor freezing-thawing process. The effective dose of hesperetin was determined (20 µM) by evaluating different concentrations of it (data is not published). All of the samples were analyzed before and also after frozen-thawed process of semen. The viability rate was evaluated by eosin/nigrosin staining, motility rate by computer-assisted sperm analyzer (CASA), the normal morphology by Diff-Quick staining, LPO by Malondialdehyde (MDA) Assay Kit, ROS levels (O2${}^{-}$ and H${}_{2}$O${}_{2}$) by DCFH-DA and DHE Kits, and the occurrence of apoptosis-like changes by Y0-PRO-1/PI Assay.

### Cryopreservation and thawing process

Following the assessment of sperm parameters, LPO, ROS, and apoptosis rates in fresh samples, the remaining volume was divided into two groups; the control group and the treatment group. Both groups were incubated for 1 hr at 37°C. Freezing process was performed according to the following steps: sperm freezing medium (Vitrolife, Goteborg, Sweden) by drop-wise on semen samples in a proportion of 1:1. After the equilibrium, at room temperature for 10 min, the cryotubes were placed in liquid nitrogen vapor (10-15 cm above the liquid nitrogen level at -80°C) for 15 min followed by plunging cryotubes in liquid nitrogen (-196) for 2 wk. Thawing process of semen samples was performed according to the following steps: the samples were removed from the liquid nitrogen and placed at room temperature for 15 min. The freezing medium was removed by adding the equal volume of Ham's F10 medium (Life global, Guelph, ON, Canada) containing 5% HAS to the semen plus freezing medium and centrifuged at 200× g for 5 min. Then, the pellet was resuspended in the same medium. Sperm parameters as well as LPO, ROS, and apoptosis rates were evaluated in post-thawed samples.

### Evaluation of ROS levels

The ROS level was measured in all three groups; fresh, control, and treatment groups using 2', 7'-dichlorofluorescin diacetate (DCFH-DA; sigma) for determining intracellular H${}_{2}$O${}_{2}$ and dihydroethidium (DHE; sigma) for determining intracellular O2${}^{-}$. Samples were incubated with DCFH-DA (25 µM) and DHE (1.25 µ) for 40 and 20 min in darkness, respectively (25, 26). DCFH-DA emitted a green fluorescent color that was evaluated at a wavelength between 530 and 500 nm (in the FL-1 channel). The DHE emitted a red fluorescent color that was evaluated at a wavelength between 590 and 700 nm (in the FL-2 channel) by flow cytometer FACScan (Becton Dickinson, San Jose, CA, USA) (25, 26).

### Evaluation of apoptosis-like changes 

The integrity of the membrane in three groups was assayed with a double-chain yo-pro-1 and propidium iodide with the Vybrant Apoptosis Assay Kit (Invitrogen, Carlsbad, CA, USA). In brief, after washing with PBS, PI (50 µg/ml) and yo-pro-1 (10 µM) were added to 1 ml of sperm suspension and incubated for 25 min at 25°C (26). Samples were evaluated using green fluorescent color for yo-pro-1 (apoptotic spermatozoa) in the FL-1channel (i.e., 530/30 band pass) and with red fluorescent color for PI (necrotic spermatozoa) in the FL-3 (i.e., 610/20 band pass) by flow cytometer FACScan (Becton Dickinson, San Jose, CA, USA). In this assay, four different spermatozoa populations can be recognized; viable spermatozoa (Yo-pro-1-/PI-), necrotic spermatozoa (Yo-pro-1-/PI+), apoptotic spermatozoa (Yo-pro-1+/PI-), and dead spermatozoa (Yo-pro-1+/PI+).

### Assessment of sperm viability

Eosin/nigrosin staining was used to determine the survival rate of sperms (27). This staining method was performed by mixing 20 µL semen with 20 µL of 1% eosin (w/v). Then, 20 µL of nigrosin (10% w/v) was added to this mixture. Sperm smear was prepared on a slide and then air dried. The sperm count was measured by observing with a light microscopy (Olympus microscope CH3O Tokyo japan) at ×1000 magnification. While the white sperm were considered as a live sperm, the colored ones (pink or red in the head area) were considered as dead. At least 200 sperm were evaluated for calculating the viability rates.

### Assessment of sperm motility rate

The parameters of motility are determined using a CASA (28). In this experiment, sperm motility was divided into four grade: (a) fast progressive, (b) slow progressive, (c) non-progressive, and (d) immotile (WHO guideline, 2010). In each group, each sample was taken at 10 μm and filled in the Makler chamber and then CASA was evaluated. The images were analyzed in five fields for each sample and the images were taken using the software video TesT sperm 3.1.

### Assessment of sperm normal morphology

The sperm morphology was determined by using Diff-Quick rapid sperm staining kit (Dayan Zist Azma Co.). At least 200 dyed sperm per each sample were counted using light microscopy (Olympus microscope CH3O Tokyo japan) at ×1000 magnification. The morphological criteria were evaluated according to the WHO guideline (29).

### Measurement of LPO level

The level of LPO was determined by the reaction of Thiobarbiuric Acid (TBA) with MDA using the MDA Assay kit (Zellbio GmbH, Deutschland) according to manufacturer's protocol. After adding the reagents to the samples, the mixture was heated for 1 hr in boiling water bath (pink color formation). Then, the pink-colored supernatant was moved into the microplate and the absorbance was read at 535 nm.

**Table 1:** Mean ± SEM percentage of total motility, progressive motility, normal morphology, and sperm concentration of sperm samples of donors


**Sperm parameters**	**Mean ± SEM**
*Concentration (×10${}^{6}$)	81.63 ± 12.62
**Total motility	70.13 ± 3.28
**Progressive motility	60.63 ± 3.05
**Normal morphology	9.56 ± 1.01
*Data presented as n (concentration); **Data presented as %	

### Ethical considerations

All patients participated in this experimental study with knowledge and consent. This study was approved by the Ethics Committee of AJA University of Medical Sciences, Iran (IR.AJAUMS.REC.1398.243).

### Statistical analysis

Data were analyzed using the SPSS (Statistical Package for the Social Sciences, version 22, SPSS Inc, Chicago, Illinois, USA). To indicate the differences between all experimental groups, one analysis of variance (one-way ANOVA) was used, followed by Tukey's post hoc test. The data were analyzed at a 95% confidence interval, and the level of significance was set at p < 0.05.

## 3. Results

### Effect of hesperetin on sperm viability

**Figure 1:**
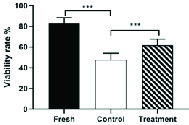
Viability rates in all three groups are indicated by eosin/nigrosin assay staining; fresh, treatment, and control groups (Mean ± SEM). *** Represent a significant difference between groups at p < 0.001 level.

By investigating the effect of hesperetin on the viability of sperm after the freezing-thawing process, we found that in the control group compared to the fresh group, the sperm viability rate decreased significantly (Table II). In addition, the viability analysis of semen samples after freezing-thawing process showed that in the tatment group, the excessive death of sperm decreased significantly compared to the control group (Table II, Figure 1).

### Effect of hesperetin on sperm motility

**Figure 2:**
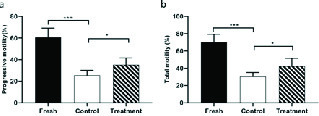
Progressive motility (a) and total motility (b) in all three groups; fresh, treatment, control groups (Mean ± SEM). ***Represent a significant difference between groups at p < 0.001 level and *Represents a significant difference between groups at p < 0.05 level. Data were compared by One-way Analysis of Variance (ANOVA) and Tukey's posttest.

The outcomes of the motility analysis demonstrated that the amount of progressive motility and total motility in the control group were reduced significantly compared to the fresh group (Table II). However, in the treatment group, hesperetin was able to prevent the reduction of progressive motility and total motility compared to the control group (Table II, Figure 2). Also, after the freezing-thawing process, the sperm velocity parameters including VSL, VAP, and VCL were significantly (Table II) reduced compared to the fresh group. While in the treatment group, hesperetin was able to increase these parameters compared to the control group (Table II, Table III).

### Effect of hesperetin on sperm morphology

**Figure 3:**
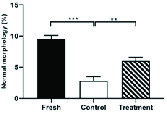
Analysis of normal morphology with Diff-Quick assay in fresh, control, and treatment groups (Mean ± SEM). ***Represent a significant difference between groups at p < 0.001 level and **Represent a significant difference between groups at p < 0.01 level. Data were compared by One-way Analysis of Variance (ANOVA) and Tukey's posttest.

By examining the abnormalities of the head, midpiece, and tail of sperm, we found that the percentage of sperm with normal morphology in the control group was significantly lower than the fresh group (Table II). However, in the treatment group, hesperetin was able to reduce the rate of sperm morphological abnormalities in comparison to the control group (Table II, Figure 3).

### Effect of hesperetin on the level of ROS 

**Figure 4:**
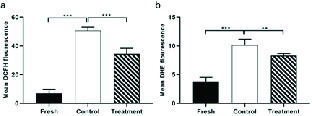
Comparison of intracellular H${}_{2}$O${}_{2}$ level (a) and intracellular O2 level (b) in all three experimental groups (Mean ± SEM). ***Represent a significant difference between groups at p < 0.001 level and **Represent a significant difference between groups at p < 0.01 level. Data were compared by One-way Analysis of Variance (ANOVA) and Tukey's posttest.

As indicated in Figure 4, the average fluorescence intensity of DCFH-DA and DHE in the control group increased significantly compared to the fresh group (Table II). Whereas, in the treatment group, hesperetin could significantly prevent the rising of the DCFH-DA and DHE in comparison to the control group (Table II).

### Effects of hesperetin on apoptotic-like change

**Figure 5:**
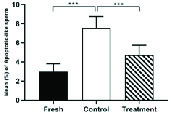
The average percentage of apoptotic-like sperm cells in fresh, control, and treatment groups (Mean ± SEM). ***Represent a significant difference between groups at p < 0.001 level. Data were compared by One-way Analysis of Variance (ANOVA) and Tukey's posttest.

The percentage of sperm cells that underwent apoptotic-like changes increased significantly in the control group compared to the fresh group following the cryopreservation process (Table II). However, in the treatment group, the amounts of these cells were significantly decreased compared to the control group (Table II, Figure 5).

### Effects of hesperetin on LPO of spermatozoa membranes

**Figure 6:**
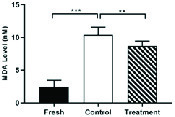
The LPO rate in the spermatozoa membrane in fresh, control, and treatment groups (Mean ± SEM). ***Represent a significant difference between groups at p < 0.001 level and **Represent a significant difference between groups at p < 0.01 level. Data were compared by One-way Analysis of Variance (ANOVA) and Tukey's posttest.

As indicated in Figure 6, in the control group, cryopreservation increased significantly the rate of LPO of the spermatozoa membrane in comparison to the fresh group (Table II). While, in the treatment group, the amount of LPO of the spermatozoa membrane were significantly decreased compared to the control group (Table II, Figure 6).

**Table 2:** Comparison all of the tests in three groups: fresh, control, and treatment groups


**Tests**	**Control group compared to the fresh group**	**P-Value**	**Treatment group compared to the control group**	**P-Value**
**Viability**	47.70 ± 2.70 vs 82.70 ± 5.66	p ≤ 0.001	61.84 ±5.88 vs 47.70 ± 6.32	p ≤ 0.001
**Progressive motility**	25.50 ± 4.59 vs 60.63 ± 3.58	p ≤ 0.001	35.25 ± 6.31 vs 25.50 ± 4.59	p = 0.021
**Total motility**	30.38 ± 4.74 vs 70.13 ± 9.28	p ≤ 0.001	42.38 ± 9.24 vs 30.38 ± 4.74	p = 0.018
**VSL,VAP,VCL parameters**	- p ≤ 0.01	- p ≤ 0.01
**Morphology**	2.87 ± 0.95 vs 9.56 ± 1.01	p ≤ 0.001	6.0. ± 0.72 vs 2.87 ± 0.95	p ≤ 0.01
**DCFH-DA **	50.75 ± 2.25 vs 7 ± 2.80	p ≤ 0.001	34.50 ± 3.89 vs 50.75 ± 2.25	p ≤ 0.001
**DHE**	10.13 ± 1.06 vs 3.81 ± 0.70	p ≤ 0.001	8.32 ± 0.36 vs 10.13 ± 1.06	p ≤ 0.01
**Apoptosis**	7.15 ± 0.67 vs 3.08 ± 0.74	p ≤ 0.001	4.45 ± 0.58 vs 7.15 ± 0.67	p ≤ 0.001
**LPO**	11.08 ± 1.76 vs 1.96 ± 0.72	p ≤ 0.001	8.23 ± 1.37 vs 11.08 ± 1.76	p ≤ 0.01
Data presented as Mean ± SD. One-way analysis of variance (ANOVA) followed by Tukey’s post test, DCFH-DA: 2’, 7’-dichlorofluorescin diacetate; DHE: Dihydroethidium; LPO: Lipid peroxidation				

**Table 3:** Comparison of human sperm velocity parameters in three groups: fresh, control, and treatment groups


**Group**	**VCL (µm/sec)**	**VSL (µm/sec)**	**VAP (µm/sec)**	**LIN (%)**	**STR (%)**	**ALH (µm)**	**WOB (%)**
**Fresh**	95.25 ± 10.34${}^{a}$	55.25 ± 13.71${}^{a}$	57.38 ± 11.36${}^{a}$	58.75 ± 14.58	95.38 ± 05.99	1.47 ± 0.78${}^{a}$	61 ± 12.02
**Control**	39.75 ± 6.69${}^{b}$	18.75 ± 7.63${}^{b}$	20.75 ± 6.81${}^{b}$	47.50 ± 19.08	87.50 ± 18.12	0.85 ± 0.21${}^{b}$	52 ± 13.30
**Treatment**	75.13 ± 10.30 ${}^{c}$	44.25 ± 8.08${}^{a}$	46.75 ± 6.79${}^{a}$	57.70 ± 09.98	94.25 ± 08.66	0.91 ± 0.18${}^{ab}$	60 ± 7.54
**P-values**	≤ 0.01	≤ 0.01	≤ 0.01	> 0.23	> 0.40	≤ 0.01	> 0.11
Data presented as Mean ± SD. One-way analysis of variance (ANOVA) followed by Tukey's post test, VCL: Velocity of curvilinear; VSL: Velocity of straight line; VAP: Velocity of average path; LIN: Linearity = VSL/VCL×100; STR: Straightness = VSL/VAP×100; ALH: Amplitude lateral head; WOB: Wobble VAP/VCL×100, A different letter in each column shows a significant difference between the groups at the level of p < 0.01and the same letter shows a non-significant difference between the groups at p > 0.05							

## 4. Discussion

In the current work, we have shown that treatment of human sperm with 20 µM hesperetin prior to the cryopreservation process can reduce the harmful effects of cryopreservation on spermatozoa and subsequently increase the fertility rate after the freezing-thawing procedure. The results of our study showed that hesperetin improved spermatozoa viability, morphology, and motility after cryopreservation. Also Hesperetin can reduce the ROS level, the rate of LPO, and percentage of like-apoptosis sperm.

Cryopreservation process of sperm is a useful and effective method for preserving the sperm of people in infertility conditions (30). Although cryopreservation has opened the promising possibility for fertility preservation, this procedure has several harmful effects on morphology, motility, and viability of sperm. Also, cryopreservation can lead to increase in sperm apoptosis rate, ROS generation, and LPO of the sperm membrane (7, 11, 31, 32).

Apoptosis induction is one of the most complex and detrimental effects of cryopreservation on spermatozoa (33). Evidence disclosed that apoptosis increases during the cryopreservation process. There are several methods to illustrate the apoptosis process. One of these methods is the yo-pro-1 assay which can detect the apoptosis changes in the sperm membrane (32). This study showed a significant increase in permeability of the sperm membrane after the freezing-thawing process. These results are consistent with the other investigations that reported the remarkable effects of cryopreservation on the sperm membranes (34). Also, the results of our study showed that hesperetin significantly reduced the percentage of apoptotic cells after the freezing process. One study showed that hesperetin significantly reduced apoptosis in doxorubicin-induced testicular cells and almost completely prevented the increase of oxidative stress (22). Hesperetin can reduce oxidative stress, inflammation, and apoptosis in diabetic rats (23). The results of the recent study on hepatocellular carcinoma cells showed that high doses of hesperetin (125, 250, and 500 μM) were used to induce apoptosis by the mitochondrial pathway (35). The cryopreservation has negative effects on different membranes existing in sperm such as acrosomal membrane, plasma membrane, and mitochondrial membrane (36). In this context, cryopreservation causes capacitation-like changes in sperm membrane and also induces the acrosomal reaction in spermatozoa (37, 38). Capacitation process and acrosomal reaction are the two most important changes in sperm membranes which are physiologically necessary for successful fertilization (39). Therefore, the effects of cryopreservation on the performance of the sperm are similar to the effects of sperm capacitation, in which both processes increase the permeability of the sperm membrane and the amount of ROS production (40).

It has been demonstrated that the level of antioxidants in the sperm decreases during cryopreservation (41). Previous studies have shown that adding antioxidants to the freezing medium can improve the quality of sperm following a cryopreservation procedure (31, 42). Accordingly, we used an antioxidant called hesperetin to reduce the harmful effects of the freezing-thawing process on spermatozoa. Hesperetin is a bioflavonoid which is used as a Chinese traditional medicine (16). Hesperetin is can be found in citrus fruits as hesperidin (its glycoside form), which acts as a prodrug (17). Recently, it has been shown that hesperetin can reduce the amount of MDA, sperm head abnormalities, and DNA damage induced by doxorubicin in rats (22). In another study, hesperetin reduced the amount of ROS in diabetic rats (23).

During the freezing-thawing process, the amount of ROS production increases significantly (41, 43). Human sperm membranes has a large amount of unsaturated fatty acids (44). ROS is a potent oxidant agents for human spermatozoa. Overproduction of ROS leads to the oxidation of unsaturated fatty acids, and subsequently increases the membrane LPO (45, 46). The results of our study showed that the level of ROS generation increased during the freezing-thawing process and 20 μM hesperetin significantly decreased ROS generation. The results of this study are consistent with recent studies showing that hesperetin has potent antioxidant effects (22, 23).

Numerous studies have shown that the freezing-thawing process of human sperm reduced significantly the total and progressive motility (47, 48), which is consistent with our study. The results of our study also showed that the freezing-thawing process reduced significantly the sperm velocity parameters including VCL, VSL, and VAP. However, in presence of 20 μM hesperetin, these parameters were significantly increased. The results of our study are also consistent with the study by Minaei and colleague who showed that Trolox, as an antioxidant, has similar effects on sperm motility (48).

## 5. Conclusion

Our findings showed that spermatozoa treatment with 20 μM hesperetin before the freezing-thawing process has protective effects against oxidative stress and could reduce the harmful effects of this process on sperm quality.

##  Conflict of Interest

The authors declare that there are no conflict of interest.

## References

[1] Sherman JK. Synopsis of the use of frozen human semen since 1964: State of the art of human semen banking. *Fertil* *Steril* 1973; 24: 397–412. 10.1016/s0015-0282(16)39678-9 4735423

[2] Stewart GJ, Cunningham AL, Driscoll GL, Tyler JP, Barr JA, Gold J, et al. Transmission of human t-cell lymphotropic virus type III (htlv-III) by artificial insemination by donor. *The Lancet* 1985; 326: 581–584. 10.1016/s0140-6736(85)90585-9 2863597

[3] Sanger WG, Olson JH, Sherman JK. Semen cryobanking for men with cancer–criteria change. *Fertil Steril *1992; 58: 1024–1027. 10.1016/s0015-0282(16)55454-5 1426353

[4] Donnelly ET, Steele EK, McClure N, Lewis SE. Assessment of DNA integrity and morphology of ejaculated spermatozoa from fertile and infertile men before and after cryopreservation. *Hum Reprod* 2001; 16: 1191–1199. 10.1093/humrep/16.6.1191 11387291

[5] Donnelly ET, McClure N, Lewis SE. Cryopreservation of human semen and prepared sperm: effects on motility parameters and DNA integrity. *Fertil Steril *2001; 76: 892–900. 10.1016/s0015-0282(01)02834-5 11704107

[6] Bollwein H, Fuchs I, Koess C. Interrelationship between plasma membrane integrity, mitochondrial membrane potential and DNA fragmentation in cryopreserved bovine spermatozoa. *Reprod Domest Anim* 2008; 43: 189–195. 10.1111/j.1439-0531.2007.00876.x 17986172

[7] O'Connell M, McClure N, Lewis SEM. The effects of cryopreservation on sperm morphology, motility and mitochondrial function. *Hum Reprod* 2002; 17: 704–709. 10.1093/humrep/17.3.704 11870124

[8] Zribi N, Feki Chakroun N, El Euch H, Gargouri J, Bahloul A, Ammar Keskes L. Effects of cryopreservation on human sperm deoxyribonucleic acid integrity. *Fertil Steril* 2010; 93: 159–166. 10.1016/j.fertnstert.2008.09.038 19027111

[9] Bansal AK, Bilaspuri GS. Impacts of oxidative stress and antioxidants on semen functions. *Vet Med Int* 2010; 2010: 686137. 1–7. 10.4061/2011/686137 PMC2943128 20871827

[10] Peris SI, Bilodeau JF, Dufour M, BaileyJL. Impact of cryopreservation and reactive oxygen species on DNA integrity, lipid peroxidation, and functional parameters in ram sperm. *Mol Reprod Dev* 2007; 74: 878–892. 10.1002/mrd.20686 17186553

[11] Wang AW, Zhang H, Ikemoto I, Anderson DJ, Loughlin KR. Reactive oxygen species generation by seminal cells during cryopreservation. *Urology* 1997; 49: 921–925. 10.1016/s0090-4295(97)00070-8 9187701

[12] Agarwal A, Makker K, Sharma R. Clinical relevance of oxidative stress in male factor infertility: An update. *Am J Reprod Immunol* 2008; 59: 2–11. 10.1111/j.1600-0897.2007.00559.x 18154591

[13] Goncalves FS, Barretto LSS, Arruda RP, Perri SHV, Mingoti GZ. Effect of antioxidants during bovine in vitro fertilization procedures on spermatozoa and embryo development. *Reprod Dom Anim* 2010; 45: 129–135. 10.1111/j.1439-0531.2008.01272.x 18992086

[14] Numan Bucak M, Sarıözkan S, Barbaros Tuncer P, Sakin F, Atessahin A, Kulaksiz R, et al. The effect of antioxidants on post-thawed Angora goat (Capra hircus ancryrensis) sperm parameters, lipid peroxidation and antioxidant activities. *Small Ruminant Research* 2010; 89: 24–30.

[15] Maxwell WMC, Watson PF. Recent progress in the preservation of ram semen. *Anim Reprod Sci* 1996; 42: 55–65.

[16] Gil-Izquierdo A, Gil MI, Ferreres F, Tomás-Barberán FA. In vitro availability of flavonoids and other phenolics in orange juice. *J Agric Food Chem* 2001; 49: 1035–1041. 10.1021/jf0000528 11262068

[17] Lee NK, Choi SH, Park SH, Park EK, Kim DH. Antiallergic activity of hesperidin is activated by intestinal microflora. *Pharmacology* 2004; 71: 174–180. 10.1159/000078083 15240993

[18] Kim HK, Jeong TS, Lee MK, Park YB, Choi MS. Lipid-lowering efficacy of hesperetin metabolites in high-cholesterol fed rats. *Clin Chim Acta* 2003; 327: 129–137. 10.1016/s0009-8981(02)00344-3 12482628

[19] Fernández-Rojas B, Gutiérrez-Venegas G. Flavonoids exert multiple periodontic benefits including anti-inflammatory, periodontal ligament-supporting, and alveolar bone-preserving effects. *Life Sci* 2018; 209: 435–454. 10.1016/j.lfs.2018.08.029 30121198

[20] Shagirtha K, Pari L. Hesperetin, a citrus flavonone, protects potentially cadmium induced oxidative testicular dysfunction in rats. *Ecotoxicol Environ Saf* 2011; 74: 2105–2111. 10.1016/j.ecoenv.2011.06.002 21719105

[21] Trivedi PP, Kushwaha S, Tripathi DN, Jena GB. Cardioprotective effects of hesperetin against doxorubicin-induced oxidative stress and DNA damage in rat. *Cardiovasc Toxicol* 2011; 11: 215–225.

[22] Trivedi PP, Tripathi DN, Jena GB. Hesperetin protects testicular toxicity of doxorubicin in rat: Role of NF j B, p38 and caspase-3. *Food Chem Toxicol* 2011; 49: 838–847. 10.1016/j.fct.2010.12.005 21168534

[23] Samie A, Sedaghat R, Baluchnejadmojarad T, Roghani M. Hesperetin, a citrus flavonoid, attenuates testicular damage in diabetic rats via inhibition of oxidative stress, inflammation, and apoptosis. *Life Sci* 2018; 210: 132–139. 10.1016/j.lfs.2018.08.074 30179627

[24] Aranganathan S, Nalini N. Efficacy of the potential chemopreventive agent, hesperetin (citrus flavanone), on 1, 2-dimethylhydrazine induced colon carcinogenesis. *Food Chem Toxicol* 2009; 47: 2594–2600. 10.1016/j.fct.2009.07.019 19632289

[25] Mahfouz R, Sharma R, Lackner J, Aziz N, Agarwal A. Evaluation of chemiluminescence and flow cytometry as tools in assessing production of hydrogen peroxide and superoxide anion in human spermatozoa. *Fertil Steril* 2009; 92: 819–827. 10.1016/j.fertnstert.2008.05.087 18710706

[26] Mahfouz RZ, du Plessis SS, Aziz N, Sharma R, Sabanegh E, Agarwal A. Sperm viability, apoptosis, and intracellular reactive oxygen species levels in human spermatozoa before and after induction of oxidative stress. *Fertil Steril *2010; 93: 814–821. 10.1016/j.fertnstert.2008.10.068 19100530

[27] Mallick Ch, Mandal S, Barik B, Bhattacharya A, Ghosh D. Protection of testicular dysfunctions by MTEC, a formulated herbal drug, in streptozotocin induced diabetic rat. *Biol Pharm Bull* 2007; 30: 84–90. 10.1248/bpb.30.84 17202665

[28] Larsen L, Scheike Th, Jensen TK, Bonde JP, Ernst E, Hjollund NH, et al. Computer-assisted semen analysis parameters as predictors for fertility of men from the general population. *Hum Reprod* 2000; 15: 1562–1567. 10.1093/humrep/15.7.1562 10875866

[29] Rowe PJ, Comhaire FH, Hargreave TB, Mahmoud AM. WHO manual for the standardised investigation, diagnosis and management of the infertile male. Cambridge: Cambridge University Press; 2000. 91.

[30] Nawroth F, Rahimi G, Isachenko E, Isachenko V, Liebermann M, Tucker MJ, et al. Cryopreservation in assisted reproductive technology: New Trends. *Semin Reprod Med* 2005; 23: 325–335. 10.1055/s-2005-923390 16317621

[31] Partyka A, Łukaszewicz E, Nizanski W. Effect of cryopreservation on sperm parameters, lipid peroxidation and antioxidant enzymes activity in fowl semen. *Theriogenology* 2012; 77: 1497–1504. 10.1016/j.theriogenology.2011.11.006 22225691

[32] Martin G, Sabido O, Durand Ph, Levy R. Cryopreservation induces an apoptosis-like mechanism in bull sperm. *Biol Reprod* 2004; 71: 28–37. 10.1095/biolreprod.103.024281 14973261

[33] Said TM, Gaglani A, Agarwal A. Implication of apoptosis in sperm cryoinjury. *Reprod Biomed Online* 2010; 21: 456–462. 10.1016/j.rbmo.2010.05.011 20800544

[34] Shabani Nashtaei M, Amidi F, Sedighi Gilani MA, Aleyasin A, Bakhshalizadeh Sh, Naji M, et al. Protective features of resveratrol on human spermatozoa cryopreservation may be mediated through 5' AMP-activated protein kinase activation. *Andrology* 2017; 5: 313–326. 10.1111/andr.12306 27992972

[35] Zhang J, Song J, Wu D, Wang J, Dong W. Hesperetin induces the apoptosis of hepatocellular carcinoma cells via mitochondrial pathway mediated by the increased intracellular reactive oxygen species, ATP and calcium. *Med Oncol *2015; 32: 101–111. 10.1007/s12032-015-0516-z 25737432

[36] Celeghini EC, de Arruda RP, de Andrade AF, Nascimento J, Raphael CF, Rodrigues PH. Effects that bovine sperm cryopreservation using two different extenders has on sperm membranes and chromatin. *Anim Reprod Sci* 2008; 104: 119–131. 10.1016/j.anireprosci.2007.02.001 17368970

[37] Watson PF. The causes of reduced fertility with cryopreserved semen. *Anim Reprod Sci* 2000; 60: 481–492. 10.1016/s0378-4320(00)00099-3 10844218

[38] Cormier N, Bailey JL. A differential mechanism is involved during heparin- and cryopreservation- induced capacitation of bovine spermatozoa. *Biol Reprod *2003; 69: 177–185. 10.1095/biolreprod.102.011056 12620931

[39] Baldi E, Luconi M, Bonaccorsi L, Muratori M, Forti G. Intracellular events and signaling pathways involved in sperm acquisition of fertilizing capacity and acrosome reaction. *Front Biosci* 2000; 3: E110–E123. 10.2741/baldi 11056077

[40] Medeiros CMO, Forell F, Oliveira ATD, Rodrigues JL. Current status of sperm cryopreservation: why isn't it better? *Theriogenology* 2002; 57: 327–344. 10.1016/s0093-691x(01)00674-4 11775978

[41] Len JS, Koh WSD, Tan SX. The roles of reactive oxygen species and antioxidants in cryopreservation. *Biosci Rep* 2019; 39: 1497–1504. 10.1042/BSR20191601 PMC6712439 31371631

[42] Banihani S, Agarwal A, Sharma R, Bayachou M. Cryoprotective effect of l-carnitine on motility, vitality and DNA oxidation of human spermatozoa. *Andrologia* 2014; 46: 637–641. 10.1111/and.12130 23822772

[43] Bucak MN, Ateşşahin A, Varışlı Ö, Yüce A, Tekin N, Akçay A. The influence of trehalose, taurine, cysteamine and hyaluronan on ram semen: Microscopic and oxidative stress parameters after freeze-thawing process. *Theriogenology* 2007; 67: 1060–1067. 10.1016/j.theriogenology.2006.12.004 17280711

[44] Craig LB, Brush RS, Sullivan MT, Zavy MT, Agbaga MP, Anderson RE. Decreased very long chain polyunsaturated fatty acids in sperm correlates with sperm quantity and quality. *J Assist Reprod Genet* 2019; 36: 1379–1385. 10.1007/s10815-019-01464-3 PMC6642247 31073727

[45] Bassiri F, Nasr-Esfahani MH, Forozanfar M, Tavalaee M. Relationship between sperm parameters with sperm function tests in infertile men with at least one failed cycle after intracytoplasmic sperm injection cycle. *Int J Fertil Steril* 2020; 13: 324–329. 10.22074/ijfs.2020.5750 PMC6875861 31710194

[46] Aitken RJ. Reactive oxygen species as mediators of sperm capacitation and pathological damage. *Mol Reprod Dev* 2017; 84: 1039–1052. 10.1002/mrd.22871 28749007

[47] Ozkavukcu S, Erdemli E, Isik A, Oztuna D, Karahuseyinoglu S. Effects of cryopreservation on sperm parameters and ultrastructural morphology of human spermatozoa. *J Assist Reprod Genet* 2008; 25: 403–411. 10.1007/s10815-008-9232-3 PMC2582121 18704674

[48] Minaei MB, Barbarestani M, Nekoonam S, Abdolvahabi MA, Takzare N, Asadi MH, et al. Effect of Trolox addition to cryopreservation media on human sperm motility. *Iran J Reprod Med* 2012; 10: 99–104. PMC4163270 25242981

